# Age-standardized incidence, prevalence, and mortality rates of autoimmune diseases in adolescents and young adults (15–39 years): an analysis based on the global burden of disease study 2021

**DOI:** 10.1186/s12889-024-19290-3

**Published:** 2024-07-05

**Authors:** Meng Zhao, Hongrui Zhai, Han Li, Feiran Wei, Hongfei Ma, Yangyang Liu, Wei Li, Pingmin Wei

**Affiliations:** 1 Department of Epidemiology and Health Statistics, School of Public Health, Southeast University, Nanjing, 210009 Jiangsu China; 2 Department of Acute Infectious Diseases, Qingdao Municipal Center for Disease Control and Prevention, Qingdao, 266033 Shandong China; 3 Key Laboratory of Environmental Medicine Engineering, School of Public Health, Ministry of Education, Southeast University, Nanjing, 210009 Jiangsu China; 4 Department of Clinical Research Center, Children’s Hospital of Nanjing Medical University, Nanjing, 210008 Jiangsu China

**Keywords:** Autoimmune disease, Epidemiology, Adolescents and young adults, Rheumatoid arthritis, Inflammatory bowel disease, Multiple sclerosis, Type 1 diabetes mellitus, Asthma, Psoriasis

## Abstract

**Background:**

Autoimmune diseases (ADs) present significant health challenges globally, especially among adolescents and young adults (AYAs) due to their unique developmental stages. Comprehensive analyses of their burden are limited. This study leverages the Global Burden of Disease (GBD) 2021 data to assess the global, regional, and national burden and trends of major ADs among AYAs from 1990 to 2021.

**Methods:**

Utilizing data from the Global Burden of Disease (GBD) Study 2021 for individuals aged 15–39 years, we employed a direct method for age standardization to calculate estimates along with 95% uncertainty intervals (UIs) for assessing the age-standardized incidence rates (ASIR), prevalence rates (ASPR), and mortality rates (ASMR) of ADs. The diseases analyzed included rheumatoid arthritis (RA), inflammatory bowel disease (IBD), multiple sclerosis (MS), type 1 diabetes mellitus (T1DM), Asthma, and Psoriasis. Trends from 1990 to 2021 were analyzed using Joinpoint regression, providing average annual percentage changes (AAPC) and 95% confidence intervals (CIs).

**Result:**

In 2021, the global ASIR, ASPR, and ASMR of RA among AYAs (per 100,000 population) were 9.46 (95% UI: 5.92 to 13.54), 104.35 (77.44 to 137.84), and 0.016 (0.013 to 0.019), respectively. For IBD, the corresponding rates were 4.08 (3.07 to 5.37), 29.55 (23.00 to 37.83), and 0.10 (0.07 to 0.12). MS exhibited rates of 1.40 (0.93 to 1.93), 16.05 (12.73 to 19.75), and 0.05 (0.04 to 0.05), respectively. T1DM had rates of 6.63 (3.08 to 11.84), 245.51 (194.21 to 307.56), and 0.54 (0.47 to 0.60). Asthma demonstrated rates of 232.22 (132.11 to 361.24), 2245.51 (1671.05 to 2917.57), and 0.89 (0.77 to 1.08). Psoriasis showed rates of 55.08 (48.53 to 61.93) and 426.16 (394.12 to 460.18) for ASIR and ASPR, respectively. From 1990 to 2021, the global ASIR of RA (AAPC = 0.47, 95% CI: 0.46 to 0.49), IBD (0.22 [0.12 to 0.33]), MS (0.22 [0.19 to 0.26]), T1DM (0.83 [0.80 to 0.86]), and Psoriasis (0.33 [0.31 to 0.34]) showed increasing trends, whereas Asthma (-0.96 [-1.03 to -0.88]) showed a decreasing trend. The global ASPR of RA (0.70 [0.68 to 0.73]), MS (0.35 [0.32 to 0.37]), T1DM (0.68 [0.66 to 0.69]), and Psoriasis (0.29 [0.27 to 0.32]) also showed increasing trends, whereas IBD (-0.20 [-0.27 to -0.13]) and Asthma (-1.25 [-1.31 to -1.19]) showed decreasing trends. Notably, the estimated global ASMR of RA (-2.35 [-2.57 to -2.12]), MS (-0.63 [-0.86 to -0.41]), T1DM (-0.35 [-0.56 to -0.14]), and Asthma (-1.35 [-1.44 to -1.26]) in AYAs declined. Additionally, the burden of disease for ADs in AYAs varies considerably across continents and between 204 countries and territories.

**Conclusion:**

ADs among AYAs present a substantial public health burden with notable regional disparities in incidence, prevalence, and mortality rates. Understanding these patterns is essential for developing targeted public health interventions and policies to mitigate the impact of ADs in this population.

**Supplementary Information:**

The online version contains supplementary material available at 10.1186/s12889-024-19290-3.

## Introduction

Autoimmune diseases (ADs) are characterized by chronic inflammatory responses resulting from immune dysregulation and loss of self-tolerance. This often leads to chronic tissue and organ damage, affecting approximately 7.6–9.4% of the global population [1]. Adolescents and young adults (AYAs), ranging from approximately 15–39 years of age, represent a population undergoing significant physical, emotional, and psychosocial changes [2–4]. Studies have indicated that ADs can pose serious health risks to AYAs [5–7]. However, previous epidemiologic studies on ADs have primarily focused on entire populations or elderly populations [8–10], with few reported in AYAs.

AYAs constitute a unique demographic likely to experience heightened physical or psychological stress compared to other age groups. This is attributed to the simultaneous occurrence of specific events in this age group, such as marriage, initiation of full-time employment, and living independently away from their parents. These factors may contribute to unhealthy conditions, making them less adherent to the treatment of their illnesses and increasing the risk of poor self-management [11]. ADs not only challenge the health of AYAs during the initial stages of the disease but also pose significant risks for long-term health consequences that can profoundly impact their lives. Rheumatoid arthritis (RA) in AYAs often leads to a lifetime burden of chronic pain and joint deformity, escalating the risk of early-onset osteoporosis and cardiovascular diseases [12]. Similarly, inflammatory bowel disease (IBD) can severely disrupt nutrient absorption, leading to growth impairments and increased susceptibility to colorectal cancer later in life [13]. Multiple sclerosis (MS), diagnosed in young adults, frequently results in progressive neurological decline, significantly reducing life expectancy and quality of life [14]. Adolescents with type 1 diabetes mellitus (T1DM) face lifelong challenges, including the risk of diabetic nephropathy and cardiovascular complications, which are major causes of morbidity and mortality in this group [15]. Asthma in AYAs can lead to chronic respiratory dysfunction, impacting physical activity and increasing the likelihood of chronic obstructive pulmonary disease in later years [16]. Psoriasis, often perceived as a skin-only disease, has systemic manifestations that include an increased risk of metabolic syndrome and cardiovascular disorders, further complicating the health landscape for affected AYAs [17]. Therefore, ADs in AYAs emerge as a significant public health issue. There is an urgent need for a comprehensive analysis and characterization of the incidence, prevalence, and mortality rates, along with their trends in recent years, in young populations worldwide. Such insights are critical for policymakers in developing primary prevention strategies.

In this study, we utilized the GBD 2021 dataset to extract global, regional (African Region, Eastern Mediterranean Region, European Region, Region of the Americas, South-East Asia Region, Western Pacific Region), and national (204 countries or territory) data about ADs (RA, IBD, MS, T1DM, Asthma, and Psoriasis) in the AYAs. Subsequently, we standardized and estimated the age-standardized incidence, prevalence, and mortality rates. Furthermore, we conducted a comprehensive analysis of the trends in these three rates over the last 30 years, aiming to provide valuable insights for the global prevention and control of ADs.

## Methods

### Data source

The Global Burden of Disease Study (GBD) is the largest and most comprehensive global observational epidemiologic survey to date. It offers a thorough assessment of health losses across 204 countries and territories, encompassing 369 diseases and injuries, as well as 88 risk factors, spanning the years 1990 to 2021 ^18, 19^.

A detailed description of the original data and methodology of GBD has been described in previous publications [18–21]. In brief, the burden of disease was estimated using a wide range of data from a representative population. These data were derived from literature reviews and identified through research collaborations, which included published scientific reports of registries and cohorts, data from cohort and registry studies, administrative health data and reports, and population surveys. DisMod-MR 2.1, an epidemiologic state-transition disease modeling software, together with MR-BRT, a Bayesian meta-regression software, were used to produce consistent disease estimates. Uncertainty intervals (UIs) were calculated from 1000 draw-levels from the posterior distribution of models, and 95% UIs were defined as the 2.5th and 97.5th values of the distribution [20, 22].

The estimates and 95% UIs (per 100,000 population) were extracted from GBD 2021 (http://ghdx.healthdata.org/gbd-results-tool). The variables included incidence, prevalence, and mortality at the global, regional, and national levels. These data were stratified by age (15–39 years), calendar year (1990–2021), causes (RA, IBD, MS, T1DM, Asthma, and Psoriasis), region (African Region, Eastern Mediterranean Region, European Region, Region of the Americas, South-East Asia Region, Western Pacific Region), and 204 countries or territories.

### Definition of ADs

ADs diagnoses and classifications in this study were based on the clinical criteria outlined by the World Health Organization (WHO), as well as the International Statistical Classification of Diseases (ISCD) and the International Classification of Diseases and Injuries (ICD-10) (https://icd.who.int/browse10/2019/en). RA, IBD, MS, T1DM, Asthma and Psoriasis are classified with the following codes according to the International Classification of Diseases (ICD-10): RA (M05-M05.9 and M08-M09.8), IBD (K50 [Crohn’s disease], K51 [Ulcerative colitis], K52 [Indeterminate colitis], K52.8[Other specified noninfective gastroenteritis and colitis], 52.9 [Noninfective gastroenteritis and colitis, unspecified]), MS (G35-G35.0), T1DM (E10-E10.11, E10.3-E10.9), Asthma (J45-J46.0), Psoriasis (L40-L41.9).

### Statistical analysis

The age-standardized rates (ASRs) estimates were calculated using the direct method of standardization and were weighted using the GBD 2021 world standard population [18, 23]. The ASRs were reported per 100,000 population with corresponding 95% UI. To further analyze the trends in age-specific autoimmune disease incidence, prevalence, and mortality rates at the global, continental, and national levels, this study employed Joinpoint regression analysis [24].

Joinpoint regression software (version 5.1.0, available at https://surveillance.cancer.gov/joinpoint/) was used to analyze trends. Joinpoint analysis facilitates the assessment of trends (1990–2021) by calculating the average annual percentage (%) change (AAPC) and its 95% confidence interval (CI). Trends were categorized as upward (AAPC > 0), downward (AAPC < 0), or stable (95% CI including 0). It is worth noting that UIs differ from CIs in that CIs can only capture uncertainty associated with sampling error, whereas UIs provide a method for propagating uncertainty from multiple sources such as sampling, model estimation, and model specification [22].

To assess the variation in ASIR, ASPR, and ASMR of major ADs across different regions and countries in 2021, we calculated the extremal quotient and coefficient of variation.

Extremal Quotient (EQ) measures the range and disparity of data between different continents and countries. The formula is as follows [25]:

$$\text{EQ}=\frac{\text{Maximum Value}}{\text{Minimum Value}}$$

Coefficient of Variation (CV) measures the relative variability of the data. The formula is as follows [26]:

$$\text{CV}=\frac{\text{Standard Deviation}}{\text{Mean}}$$

By calculating the ratio of the standard deviation to the mean for each disease and each metric, we can assess the relative variability of the data across different continents and countries.

Data collation and visualizations were conducted using R software (Version 4.3.1). A threshold of 0.05 for the two-tailed *P*-value was employed to establish statistical significance.

## Results

### Rheumatoid arthritis

**Fig. 1:**
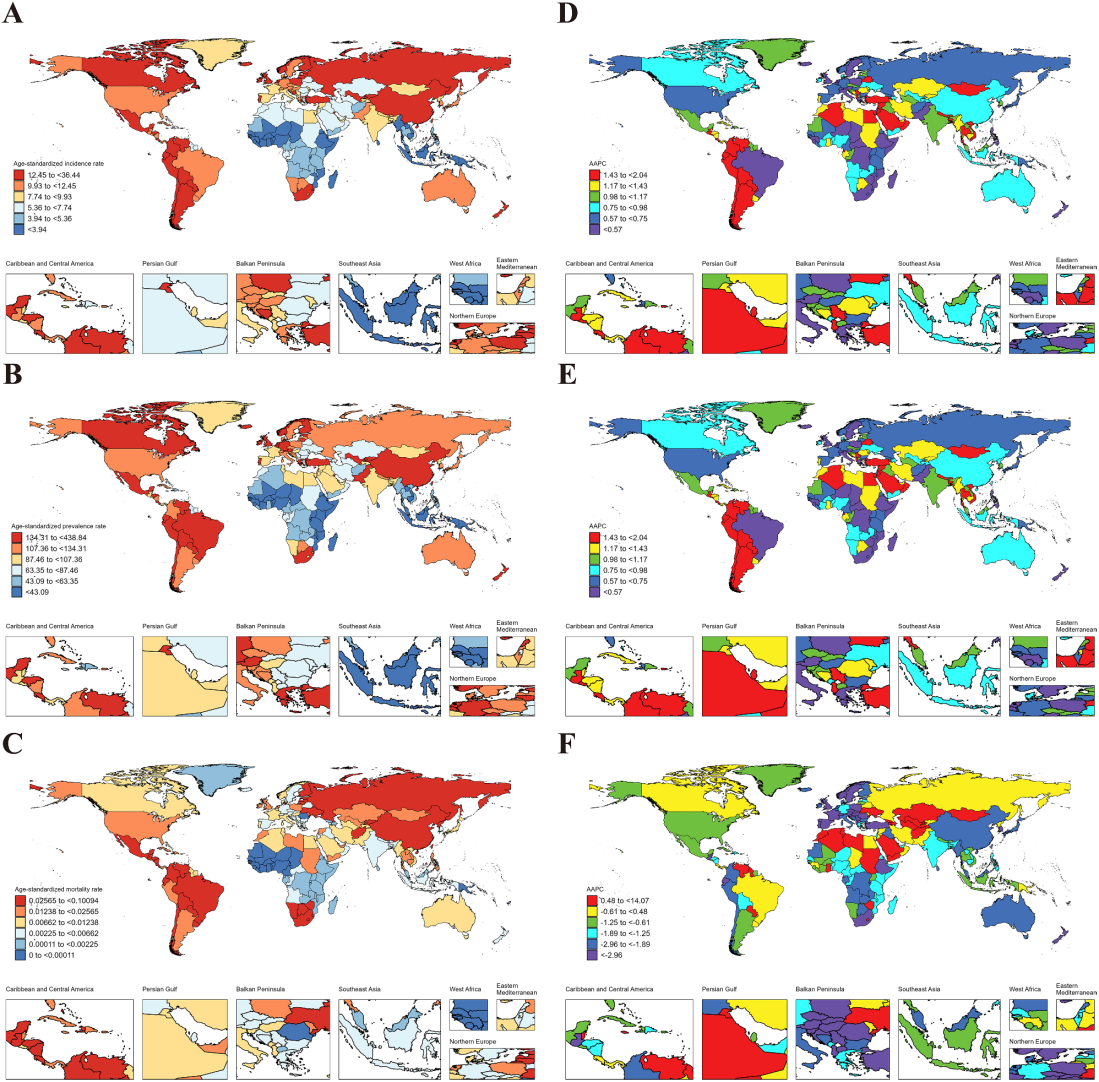
ASIR, ASPR and ASMR of RA in AYAs at the national level in 2021 (**A**, **B**, **C**), and their changing trends from 1990 to 2021 (**D**, **E**, **F**)

In 2021, the global age-standardized incidence rate (ASIR), age-standardized prevalence rate (ASPR), and age-standardized mortality rate (ASMR) in AYAs were 9.46 (95% UI: 5.92 to 13.54), 104.35 (77.44 to 137.84) and 0.016 (0.013 to 0.019), respectively (Table 1). At the regional level, the Region of the Americas exhibited the highest ASIR (14.84 [9.89 to 20.49]), ASPR (160.93 [125.21 to 202.99]), and ASMR (0.035 [0.031 to 0.041]); the lowest ASIR (5.21 [3.19 to 7.60]) and ASPR (55.64 [40.07 to 74.69]) were noticed in African Region; and the lowest ASMR were found in South-East Asia Region (0.006 [0.004 to 0.010]) (Table 1). On the national level, variations in ASIR, ASPR, and ASMR were observed across 204 countries and territories (Fig. 1. A, B, C). Among them, Peru (36.44 [23.53 to 52.10]), Mexico (26.35 [17.17 to 36.65]), and South Africa (23.06 [15.95 to 31.26]) showed the highest ASIR, while the lowest ASIR were in Indonesia (2.54 [1.38 to 4.02]), Malaysia (2.66 [1.44 to 4.26]), and Chad (2.84 [1.59 to 4.38]). For ASPR, Peru (438.84 [335.86 to 561.45]) retained the top position, followed by Kuwait (260.52 [197.30 to 328.99]), and South Africa (228.43 [175.43 to 290.36]); while the lowest ASPR were observed in Indonesia (30.26 [19.97 to 43.12]), Malaysia (32.13 [21.57 to 45.62]), and Chad (32.17 [21.94 to 44.93]). In terms of ASMR, Mexico (0.10 [0.08 to 0.13]) emerged as the country/region with the highest rate, followed by Lithuania (0.08 [0.07 to 0.10]), and Venezuela (0.08 [0.05 to 0.12]); while the lowest ASMR were in Cabo Verde (5.62 × 10^− 7^ [1.10 × 10^− 7^ to 3.75 × 10^− 6^]), Mauritania (1.34 × 10^− 6^ [3.06 × 10^− 7^ to 7.11 × 10^− 6^]), and Sao Tome and Principe (1.37 × 10^− 6^ [3.27 × 10^− 7^ to 7.94 × 10^− 6^]) (Fig. 1. A, B, C & Table S4-6).

**Table 1:** ASIR, ASPR and ASMR of autoimmune diseases in adolescents and young adults at the global and regional levels in 2021

Characteristics	Location	RA (95% UI)	IBD (95% UI)	MS (95% UI)	T1DM (95% UI)	Asthma (95% UI)	Psoriasis (95% UI)
ASIR	Global	9.46 (5.92 to 13.54)	4.08 (3.07 to 5.37)	1.40 (0.93 to 1.93)	6.63 (3.08 to 11.84)	232.22 (132.11 to 361.24)	55.08 (48.53 to 61.93)
	African Region	5.21 (3.19 to 7.60)	1.55 (1.15 to 2.09)	0.65 (0.38 to 0.96)	4.85 (2.14 to 8.76)	257.41 (155.46 to 375.82)	33.44 (29.24 to 37.79)
	Eastern Mediterranean Region	8.29 (5.32 to 11.79)	3.79 (2.82 to 5.08)	2.23 (1.37 to 3.24)	7.20 (3.20 to 13.02)	231.75 (140.11 to 348.76)	55.55 (48.70 to 62.83)
	European Region	12.09 (7.59 to 17.26)	9.43 (7.15 to 12.32)	4.25 (2.94 to 5.77)	12.05 (6.17 to 20.18)	312.43 (166.90 to 508.17)	82.19 (72.16 to 92.47)
	Region of the Americas	14.84 (9.89 to 20.49)	7.32 (5.59 to 9.47)	3.18 (2.30 to 4.12)	9.67 (4.85 to 16.65)	416.23 (222.15 to 674.32)	74.54 (65.80 to 83.76)
	South-East Asia Region	7.37 (4.37 to 11.08)	3.95 (2.92 to 5.33)	0.56 (0.32 to 0.85)	6.26 (2.66 to 11.74)	150.87 (88.73 to 232.94)	50.13 (44.17 to 56.24)
	Western Pacific Region	11.15 (6.77 to 16.34)	1.77 (1.32 to 2.36)	0.30 (0.17 to 0.45)	3.95 (1.77 to 7.17)	178.92 (96.50 to 288.77)	52.10 (45.88 to 58.68)
ASPR	Global	104.35 (77.44 to 137.84)	29.55 (23.00 to 37.83)	16.05 (12.73 to 19.75)	245.51 (194.21 to 307.56)	2245.51 (1671.05 to 2917.57)	426.16 (394.12 to 460.18)
	African Region	55.64 (40.07 to 74.69)	11.12 (8.59 to 14.50)	7.08 (5.08 to 9.44)	202.70 (165.21 to 249.11)	2230.59 (1739.72 to 2780.63)	260.36 (239.97 to 282.02)
	Eastern Mediterranean Region	104.00 (77.17 to 135.36)	27.59 (21.34 to 35.95)	25.55 (18.98 to 32.89)	278.00 (214.61 to 349.31)	1878.94 (1407.89 to 2428.17)	395.23 (363.68 to 429.14)
	European Region	129.17 (95.87 to 170.58)	78.39 (61.70 to 100.07)	49.01 (39.59 to 59.45)	418.80 (337.09 to 513.20)	4250.05 (3034.34 to 5691.86)	725.76 (673.79 to 780.33)
	Region of the Americas	160.93 (125.21 to 202.99)	52.39 (40.96 to 66.86)	35.62 (30.31 to 41.27)	367.95 (298.94 to 446.92)	4340.40 (3177.27 to 5734.92)	646.25 (601.34 to 692.93)
	South-East Asia Region	80.62 (57.41 to 110.99)	25.18 (19.25 to 33.11)	6.11 (4.31 to 8.31)	218.89 (166.50 to 288.08)	1233.42 (919.51 to 1598.10)	364.69 (335.92 to 395.07)
	Western Pacific Region	120.33 (87.11 to 161.86)	12.20 (9.45 to 15.81)	3.39 (2.36 to 4.64)	141.98 (109.99 to 182.40)	1617.82 (1128.71 to 2214.79)	369.98 (340.78 to 401.18)
ASMR	Global	0.016 (0.013 to 0.019)	0.098 (0.072 to 0.117)	0.047 (0.040 to 0.055)	0.537 (0.471 to 0.602)	0.892 (0.766 to 1.076)	——
	African Region	0.007 (0.005 to 0.016)	0.258 (0.135 to 0.371)	0.077 (0.047 to 0.113)	0.619 (0.469 to 0.786)	1.798 (1.404 to 2.429)	——
	Eastern Mediterranean Region	0.011 (0.007 to 0.018)	0.068 (0.045 to 0.104)	0.053 (0.040 to 0.067)	0.677 (0.521 to 0.835)	0.856 (0.672 to 1.154)	——
	European Region	0.020 (0.018 to 0.022)	0.099 (0.092 to 0.106)	0.128 (0.118 to 0.139)	0.444 (0.418 to 0.469)	0.161 (0.150 to 0.176)	——
	Region of the Americas	0.035 (0.031 to 0.041)	0.112 (0.107 to 0.118)	0.081 (0.077 to 0.080)	0.766 (0.728 to 0.807)	0.380 (0.351 to 0.419)	——
	South-East Asia Region	0.006 (0.004 to 0.010)	0.074 (0.053 to 0.106)	0.008 (0.004 to 0.009)	0.580 (0.475 to 0.687)	1.385 (1.149 to 1.783)	——
	Western Pacific Region	0.022 (0.014 to 0.030)	0.032 (0.025 to 0.042)	0.007 (0.006 to 0.008)	0.261 (0.220 to 0.311)	0.380 (0.333 to 0.442)	——

Note: ASIR, age-standardized incidence rate; ASPR, age-standardized prevalence rate; ASMR, age-standardized mortality rate; RA, rheumatoid arthritis; IBD, inflammatory bowel disease; MS, multiple sclerosis; T1DM, type 1 diabetes mellitus; UI Uncertainty Interval

**Fig. 2:**
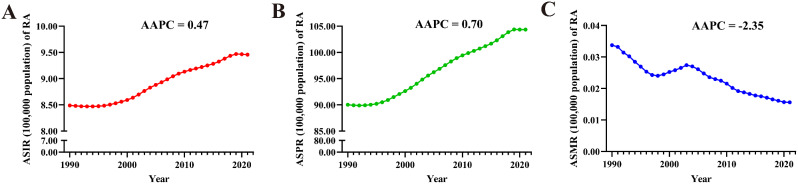
Trends in global RA burden from 1990 to 2021. A: ASIR, B: ASPR, C: ASMR

From 1990 to 2021, ASIR (AAPC = 0.47 [95% CI: 0.46 to 0.49]) and ASPR (0.70 [0.68 to 0.73]) of RA in AYAs globally exhibited an upward trend. In contrast, the ASMR had a downward trend (-2.35 [-2.57 to -2.12]) (Fig. 2). At the regional level, the South-East Asia Region had the largest increase in ASIR (1.16 [1.12 to 1.19]) and ASPR (1.23 [1.19 to 1.26]) for RA in AYAs; while the African Region experienced the most significant decrease in ASMR (-4.15 [-4.87 to -3.43]) (Table 2). On a national level, the largest increase in ASIR was observed in Oman (2.04 [1.96 to 2.12]), followed by Saudi Arabia (1.88 [1.81 to 1.96]), and Chile (1.83 [1.79 to 1.87]), while the largest decreases occurred in Taiwan (Province of China) (-0.44 [-0.57 to -0.31]), South Africa (-0.42 [-0.44 to -0.39]), and Philippines (-0.36 [-0.48 to -0.24]). The largest increase in ASPR was in Saudi Arabia (2.44 [2.36 to 2.51]), followed by Oman (2.39 (2.34 to 2.44), and Kuwait (2.12 [2.04 to 2.20]); while the largest decreases were noticed in Philippines (-0.25 [-0.30 to -0.20]), Taiwan (Province of China) (-0.20 [-0.33 to -0.07]), and Sweden (-0.13 [-0.19 to -0.07]). The countries with the largest decrease in the ASMR of RA in AYAs were Slovenia (-5.58 [-6.82 to -4.33]), Norway (-5.14 [-7.47 to -2.75]), and Hungary (-4.90 [-5.53 to -4.27]). Notably, 31 countries had statistically significant increases in ASMR (AAPC > 0 and *P* < 0.05), with the largest increase in Turkmenistan (14.07 [8.87 to 19.51]), followed by Mauritius (11.03 [0.17 to 23.06]), and Kazakhstan (9.36 [7.07 to 11.71]) (Fig. 1.D, E, F and Tables S7-9).

**Table 2:** Changing trends of ASIR, ASPR and ASMR of autoimmune diseases in adolescents and young adults at the regional levels from 1990 to 2021

Characteristics	Location	RA	IBD	MS	T1DM	Asthma	Psoriasis
		AAPC (95% CI)	AAPC (95% CI)	AAPC (95% CI)	AAPC (95% CI)	AAPC (95% CI)	AAPC (95% CI)
ASIR	African Region	-0.18 (-0.21 to -0.15) *	0.45 (0.42 to 0.47) *	0.43 (0.40 to 0.46) *	0.13 (0.09 to 0.16) *	-0.80 (-0.85 to -0.74) *	0.28 (0.26 to 0.30) *
	Eastern Mediterranean Region	0.76 (0.72 to 0.81) *	0.76 (0.72 to 0.80) *	0.83 (0.76 to 0.89) *	0.47 (0.42 to 0.52) *	-0.99 (-1.04 to -0.95) *	0.63 (0.60 to 0.65) *
	European Region	0.80 (0.77 to 0.82) *	0.29 (0.24 to 0.33) *	0.66 (0.64 to 0.69) *	1.51 (1.46 to 1.56) *	-1.43 (-1.54 to -1.31) *	0.41 (0.34 to 0.48) *
	Region of the Americas	0.95 (0.92 to 0.99) *	-0.42 (-0.50 to -0.33) *	-0.04 (-0.10 to 0.02) *	0.58 (0.52 to 0.65) *	-0.42 (-0.64 to -0.21) *	0.21 (0.17 to 0.25) *
	South-East Asia Region	1.16 (1.12 to 1.19) *	0.89 (0.53 to 1.25) *	0.61 (0.60 to 0.62) *	0.31 (0.26 to 0.36) *	-1.04 (-1.09 to -0.98) *	0.36 (0.32 to 0.39) *
	Western Pacific Region	0.62 (0.57 to 0.67) *	1.77 (1.41 to 2.12) *	0.75 (0.59 to 0.91) *	1.71 (1.62 to 1.80) *	-1.13 (-1.20 to -1.05) *	0.91 (0.86 to 0.97) *
ASPR	African Region	0.01 (-0.01 to 0.03) *	0.41 (0.40 to 0.42) *	0.54 (0.53 to 0.56) *	0.01 (-0.01 to 0.02) *	-1.09 (-1.16 to -1.01) *	0.40 (0.38 to 0.42) *
	Eastern Mediterranean Region	1.07 (1.04 to 1.11) *	0.73 (0.65 to 0.81) *	1.18 (1.10 to 1.25) *	0.79 (0.77 to 0.80) *	-1.07 (-1.13 to -1.02) *	0.83 (0.80 to 0.85) *
	European Region	0.96 (0.93 to 0.99) *	0.05 (0.00 to 0.11) *	0.91 (0.88 to 0.94) *	1.55 (1.52 to 1.59) *	-1.34 (-1.39 to -1.29) *	0.47 (0.45 to 0.49) *
	Region of the Americas	0.92 (0.90 to 0.95) *	-0.97 (-1.09 to -0.84) *	-0.05 (-0.09 to -0.02) *	0.30 (0.27 to 0.34) *	-0.34 (-0.46 to -0.22) *	0.13 (0.12 to 0.14) *
	South-East Asia Region	1.23 (1.19 to 1.26) *	0.87 (0.50 to 1.24) *	0.72 (0.70 to 0.73) *	0.31 (0.27 to 0.34) *	-1.79 (-1.86 to -1.72) *	0.34 (0.29 to 0.39) *
	Western Pacific Region	1.08 (1.00 to 1.15) *	1.22 (0.94 to 1.50) *	1.14 (0.99 to 1.29) *	1.00 (0.95 to 1.05) *	-1.17 (-1.21 to -1.12) *	0.91 (0.89 to 0.94) *
ASMR	African Region	-4.15 (-4.87 to -3.43) *	0.68 (0.56 to 0.80) *	1.95 (1.78 to 2.13) *	-0.60 (-0.68 to -0.52) *	-1.63 (-1.80 to -1.46) *	——
	Eastern Mediterranean Region	0.64 (0.39 to 0.89) *	-0.02 (-0.14 to 0.10) *	2.20 (2.05 to 2.35) *	0.55 (0.33 to 0.77) *	-1.56 (-1.67 to -1.44) *	——
	European Region	-1.58 (-2.27 to -0.89) *	-0.67 (-1.17 to -0.18) *	-1.77 (-2.21 to -1.33) *	-1.01 (-1.42 to -0.60) *	-4.18 (-4.37 to -4.00) *	——
	Region of the Americas	-0.38 (-0.92 to 0.15) *	0.54 (0.08 to 1.01) *	-0.03 (-0.25 to 0.19) *	0.74 (0.51 to 0.97) *	-1.52 (-1.90 to -1.14) *	——
	South-East Asia Region	-1.39 (-1.81 to -0.97) *	-1.34 (-2.15 to -0.52) *	2.35 (2.01 to 2.68) *	-0.97 (-1.41 to -0.54) *	-2.12 (-2.49 to -1.74) *	——
	Western Pacific Region	-2.59 (-2.96 to -2.23) *	-1.90 (-2.16 to -1.63) *	1.17 (0.90 to 1.44) *	-1.29 (-1.61 to -0.97) *	-1.84 (-1.95 to -1.73) *	——

Note: *Significantly different from 0 at alpha = 0.05 (*P* < 0.05). RA, rheumatoid arthritis; IBD, inflammatory bowel disease; MS, multiple sclerosis; T1DM, type 1 diabetes mellitus; AAPC, average annual percentage change; CI, confidence interval

### Inflammatory bowel disease

**Fig. 3:**
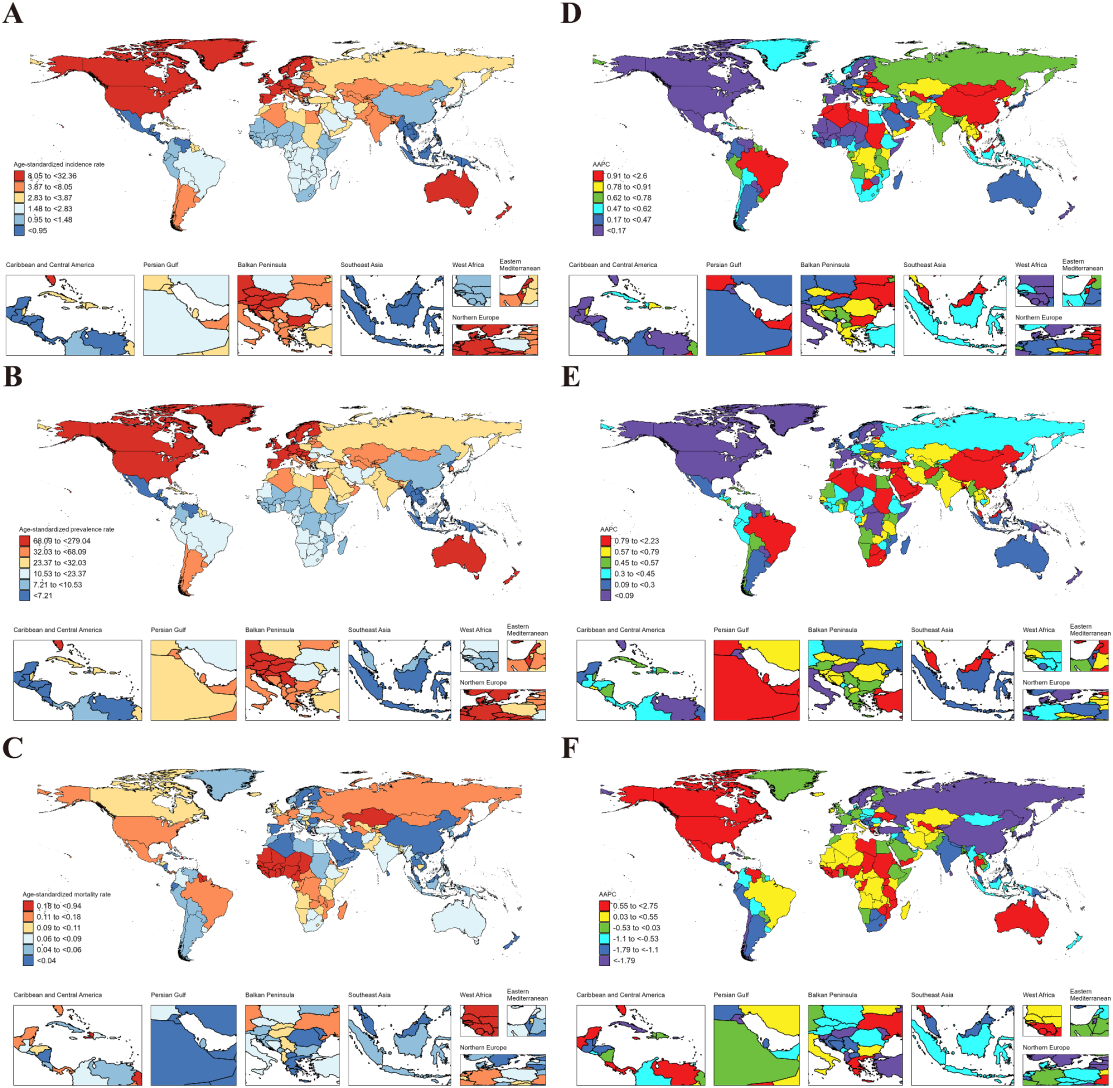
ASIR, ASPR and ASMR of IBD in AYAs at the national level in 2021 (**A**, **B**, **C**), and their changing trends from 1990 to 2021 (**D**, **E**, **F**)

In 2021, from a global perspective, ASIR, ASPR, ASMR for IBD in AYAs were 4.08 (95% UI: 3.07 to 5.37), 29.55 (23.00 to 37.83), and 0.10 (0.07 to 0.12), respectively (Table 1). At the regional level, European Region exhibited the highest ASIR (9.43 [7.15 to 12.32]) and ASPR (78.39 [61.70 to 100.07]) for IBD among AYAs worldwide; the lowest ASIR (1.55 [1.15 to 2.09]) and ASPR (11.12 [8.59 to 14.50]) were observed in African Region. Meanwhile, the African Region (0.258 [0.135 to 0.371]) had the highest ASMR, and Western Pacific Region (0.032 [0.025 to 0.042]) had the lowest ASMR (Table 1). At the national level, Canada (32.36 [24.81 to 42.12]) had the highest ASIR of IBD among AYAs globally, followed by Greenland (29.66 [22.97 to 38.35]), and the Netherlands (27.85 [21.36 to 35.68]), while the lowest were in Mexico (0.19 [0.14 to 0.28]), Philippines (0.55 [0.39 to 0.76]), El Salvador (0.58 [0.40 to 0.80]). For ASPR, Canada retained the top position (279.04 [212.09 to 365.49]), followed by Netherlands (246.48 [196.19 to 313.18]), and San Marino (241.12 [191.34 to 313.18]); while the lowest ASPR were Mexico (1.77 [1.28 to 2.43]), Philippines (4.02 [2.98 to 5.44]), and Guatemala (4.49 [3.23 to 6.20]). Regarding ASMR, Guinea-Bissau (0.94 [0.43 to 1.74]) emerged as the country/region with the highest rate, followed by the Gambia (0.84 [0.38 to 1.69]) and Mali (0.84 [0.39 to 1.59]). The lowest ASMR were in Singapore (0.005 [0.004 to 0.006]), Sri Lanka (0.005 [0.003 to 0.009]), and Malaysia (0.011 [0.006 to 0.018]) (Fig. 3. A, B, C & Table S4-6).

**Fig. 4:**
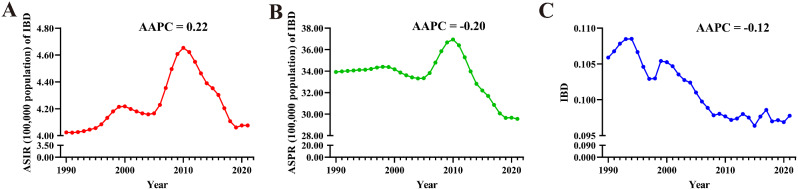
Trends in global IBD burden from 1990 to 2021. A: ASIR, B: ASPR, C: ASMR

From 1990 to 2021, the global ASIR (AAPC = 0.22 [95% CI: 0.12 to 0.33]) of IBD in AYAs exhibited a significant downward trend, while the ASPR (-0.20 [-0.27 to -0.13]) indicated a downward trend. No statistically significant trends were observed for ASMR (-0.12 [-0.33 to 0.10]) (Fig. 4). At the regional level, the Western Pacific Region (1.77 [1.41 to 2.12]) experienced the largest increase in ASIR for IBD in AYAs, while the largest decrease occurred in the Region of the Americas (-0.42 [-0.50 to -0.33]). The Western Pacific Region had the largest increase in ASPR (1.22 [0.94 to 1.50]), while the Region of the Americas (-0.97 [-1.09 to -0.84]) decreased the most. Conversely, the region with the largest increase in ASMR was African Region (0.68 [0.56 to 0.80]), while the largest decrease occurred in Western Pacific Region (-1.90 [-2.16 to -1.63]) (Table 2). At the national level, China (2.60 [1.98 to 3.22]) witnessed the largest increase in ASIR for IBD in AYAs, followed by Libya (2.59 [2.35 to 2.82]), and Oman (1.56 [1.51 to 1.62]); while the largest decreases occurred in Italy (-0.76 [-0.85 to -0.67]), Finland (-0.69 [-0.85 to -0.53]), and Denmark (-0.54 [-0.78 to -0.30]). Libya (2.23 [1.92 to 2.55]) had the largest increase in ASPR, followed by China (1.85 [1.28 to 2.43]) and Maldives (1.52 [1.47 to 1.58]). The largest decreases were noticed in the Canada (-1.56 [-1.67 to -1.46]), Italy (-1.29 [-1.36 to -1.22]), and Iceland (-0.87 [-0.98 to -0.77]). Regarding ASMR, Australia (2.12 [0.18 to 4.08]) demonstrated the largest increase, followed by Thailand (1.66 [0.43 to 2.89]) and Zimbabwe (1.62 [1.24 to 2.00]). The largest decreases occurred in Estonia (-6.83 [-8.41 to -5.22]), Lithuania (-5.56 [-7.00 to -4.09]), and Latvia (-5.39 [-6.64 to -4.11]). It is worth noting that 42 countries had statistically significant increases in ASMR (AAPC > 0 and *P* < 0.05) (Fig. 3. D, E, F, and Table S7-9).

### Multiple sclerosis

**Fig. 5:**
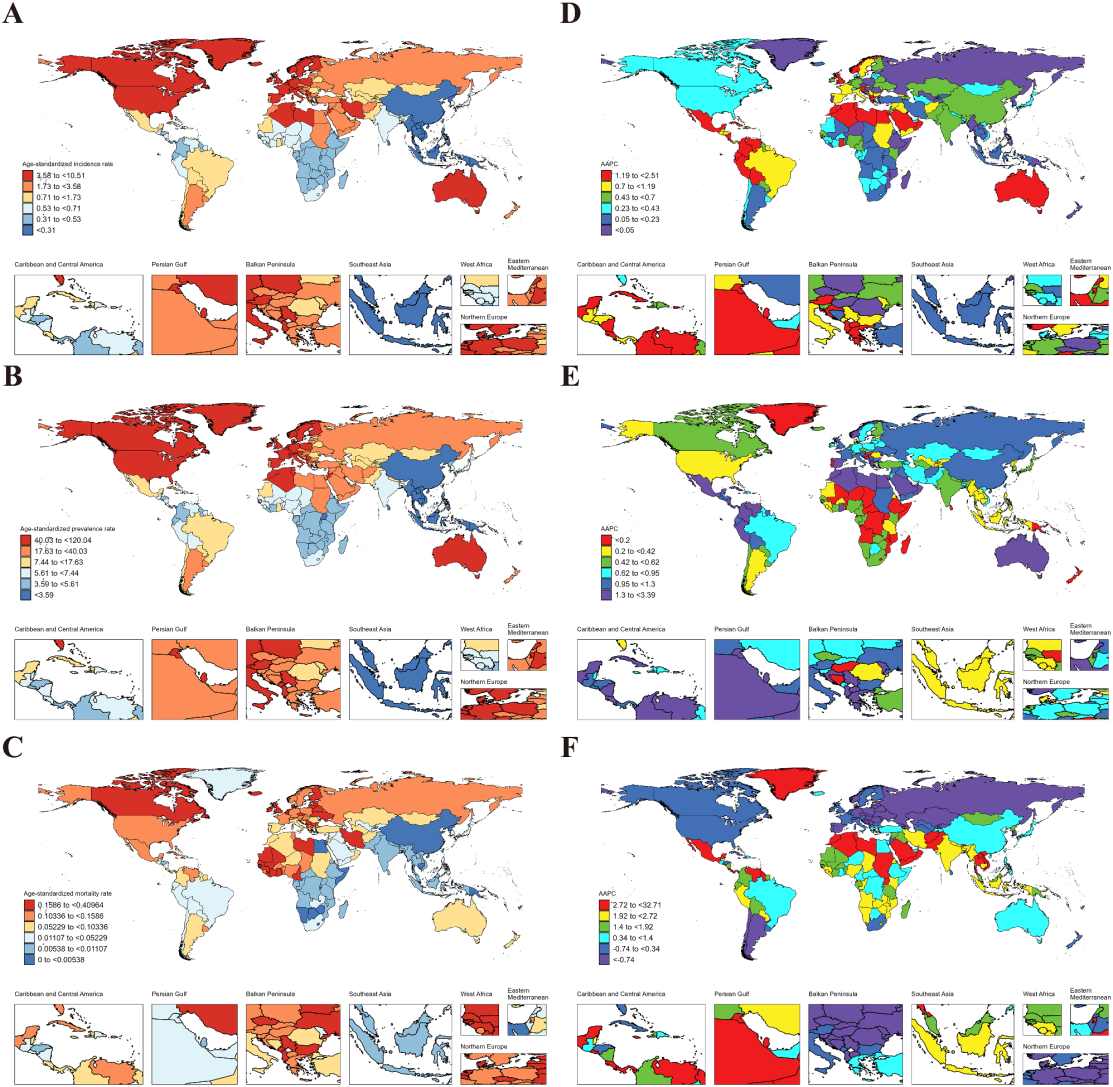
ASIR, ASPR and ASMR of MS in AYAs at the national level in 2021 (**A**, **B**, **C**), and their changing trends from 1990 to 2021 (**D**, **E**, **F**)

In 2021, the ASIR, ASPR, and ASMR of MS in global AYAs were 1.40 (95% UI: 0.93 to 1.93), 16.05 (12.73 to 19.75), and 0.05 (0.04 to 0.05), respectively (Table 1). At the regional level, the European Region exhibited the highest ASIR (4.25 [2.94 to 5.77]), ASPR (49.01 [39.59 to 59.45]), and ASMR (0.128 [0.118 to 0.139]) for MS in global AYAs. The lowest ASIR (0.30 [0.17 to 0.45]), ASPR (3.39 [2.36 to 4.64]), and ASMR (0.007 [0.006 to 0.008]) were observed in Western Pacific Region (Table 1). On the national level, Sweden (10.51 [6.75 to 14.95]) had the highest ASIR for MS in global AYAs, followed by Ireland (9.81 [6.22 to 14.41]) and Canada (9.41 [8.16 to 10.72]), while the lowest were in Maldives (0.15 [0.08 to 0.25]), Papua New Guinea (0.162 [0.083 to 0.270]), and Nauru (0.165 [0.085 to 0.276]). Sweden (120.04 [91.15 to 151.22]) had the highest ASPR for MS in global AYAs, followed by Ireland (111.86 [82.17 to 146.51]) and Canada (102.10 [95.20 to 109.06]), while the lowest ASPR were in Nauru (1.74 [1.09 to 2.59]), Papua New Guinea (1.74 [1.11 to 2.61]), and Maldives (1.78 [1.15 to 2.60]). Regarding ASMR, Albania (0.41 [0.18 to 0.80]) ranked the highest, followed by Ukraine (0.27 [0.17 to 0.38]) and United Kingdom (0.27 [0.25 to 0.28]), while the lowest ASMR were in Zimbabwe (1.09 × 10^− 5^ [4.29 × 10^− 6^ to 2.34 × 10^− 5^]), San Marino (1.27 × 10^− 5^ [3.02 × 10^− 6^ to 3.27 × 10^− 5^]), and Northern Mariana Islands (1.50 × 10^− 5^ [3.69 × 10^− 6^ to 3.96 × 10^− 5^]) (Fig. 5. A, B, C & Table S4-6).

**Fig. 6:**
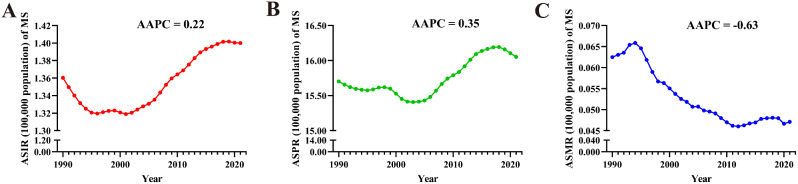
Trends in global MS burden from 1990 to 2021. A: ASIR, B: ASPR, C: ASMR

From 1990 to 2021, the global ASIR (AAPC = 0.22 [95% CI: 0.19 to 0.26]), ASPR (0.35 [0.32 to 0.37]), of MS in AYAs globally exhibited an upward trend. In contrast, ASMR had a downward trend (-0.63 [-0.86 to -0.41]) (Fig. 6). At the regional level, the Eastern Mediterranean Region experienced the largest increase in ASIR for MS in AYAs (0.83 [0.76 to 0.89]). Meanwhile, Eastern Mediterranean Region retained the top position for the largest increase in ASPR (1.18 [1.10 to 1.25]), while Region of the Americas (-0.05 [-0.09 to -0.02]) decreased the most. The region with the largest increase in ASMR was the South-East Asia Region (2.35 [2.01 to 2.68]), while the largest decrease occurred in European Region (-1.77 [-2.21 to -1.33]) (Table 2). On the national level, Egypt (2.51 [2.33 to 2.70]) witnessed the largest increase in ASIR for AYAs with MS, followed by Taiwan (Province of China) (2.39 [2.34 to 2.43]) and Oman (1.94 [1.91 to 1.97]). The largest decreases occurred in Hungary (-0.85 [-0.93 to -0.77]), Ethiopia (-0.25 [-0.28 to -0.21]), and Romania (-0.23 [-0.27 to -0.20]). Additionally, Taiwan (Province of China) (3.39 [3.32 to 3.47]) had the largest increase in ASPR, followed by Egypt (2.77 [2.59 to 2.96]) and Kuwait (2.55 [2.43 to 2.67]). The largest decreases were noticed in Hungary (-0.23 [-0.26 to -0.20]), Somalia (-0.20 [-0.30 to -0.09]), and Ethiopia (-0.14 [-0.18 to -0.10]). Regarding ASMR, Mauritius (32.71 [30.61 to 34.83]) demonstrated the largest increase, followed by Kuwait (24.82 [20.74 to 29.03]) and Bahrain (10.35 [9.09 to 11.63]). The largest decrease occurred in Estonia (-4.08 [-5.27 to -2.88]), Poland (-3.62 [-4.32 to -2.91]), and Slovenia (-3.51 [-5.43 to -1.55]) (Fig. 2. D, E, F and Table S7-9). It is worth noting that 130 countries were in a state of increasing ASMR (AAPC > 0 and *P* < 0.05) (Fig. 5. D, E, F and Tables S7-9).

### Type 1 diabetes mellitus

**Fig. 7:**
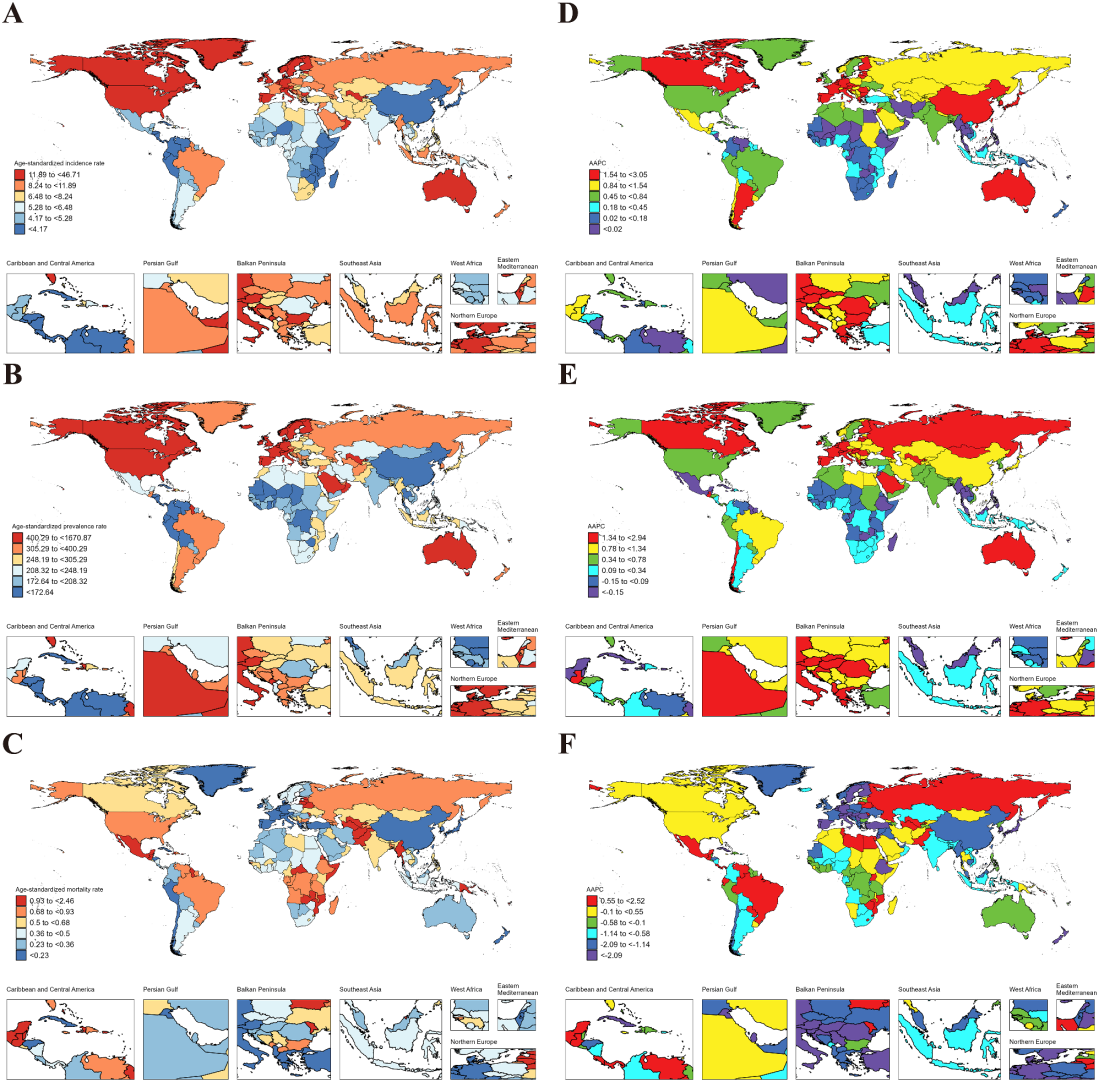
ASIR, ASPR and ASMR of T1DM in AYAs at the national level in 2021 (**A**, **B**, **C**), and their changing trends from 1990 to 2021 (**D**, **E**, **F**)

In 2021, the ASIR, ASPR, and ASMR of T1DM in global AYAs were 6.63 (95% UI: 3.08 to 11.84), 245.51 (194.21 to 307.56), and 0.54 (0.47 to 0.60), respectively (Table 1). At the regional level, the European Region exhibited the highest ASIR (12.05 [6.17 to 20.18]) and ASPR (418.80 [337.09 to 513.20]) for T1DM in global AYAs, while the lowest ASIR (3.95 [1.77 to 7.17]) and ASPR (141.98 [109.99 to 182.40]) were observed in the Western Pacific Region. In terms of ASMR, the Region of the Americas had the highest rate (0.766 [0.728 to 0.807]); and the Western Pacific Region (0.261 [0.220 to 0.311]) had the lowest ASMR (Table 1). On the national level, Finland (46.71 [39.26 to 55.19]) had the highest ASIR for T1DM in global AYAs, followed by Canada (34.93 [26.66 to 44.14]) and Italy (31.68 [13.19 to 58.02]), while the lowest ASIR were in Costa Rica (2.00 [0.92 to 3.61]), Venezuela (Bolivarian Republic of) (2.17 [0.96 to 3.94]), Colombia (2.29 [1.09 to 3.98]). Similarly, Finland (1670.87 [1589.13 to 1754.54]) had the highest ASPR for T1DM in global AYAs, followed by Canada (1311.35 [1197.80 to 1424.62]) and Italy (930.32 [694.37 to 1243.98]), while the lowest ASPR were in Costa Rica (89.38 [69.63 to 114.00]), China (95.90 [71.86 to 127.26]), and Colombia (96.67 [76.64 to 122.17]). Regarding ASMR, Haiti (2.46 [1.32 to 4.23]) had the highest rate, followed by Mauritius (2.03 [1.67 to 2.38]) and Trinidad and Tobago (2.02 [1.48 to 2.63]), while the lowest ASMR were in Singapore (0.045 [0.038 to 0.053]), Italy (0.083 [95% UI: 0.079 to 0.087]), and Greece (0.09 [0.08 to 0.11]) (Fig. 7. A, B, C & Table S4-6).

**Fig. 8:**
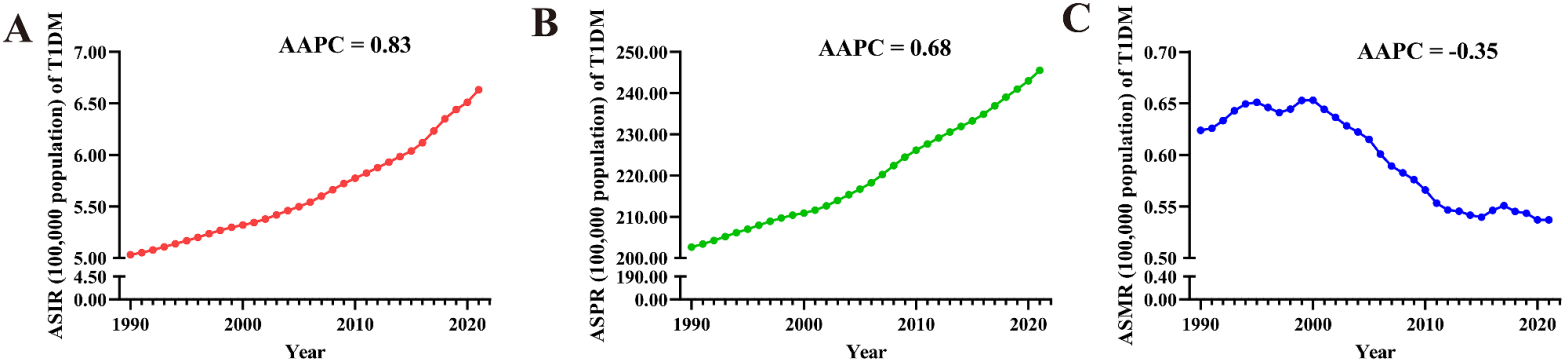
Trends in global T1DM burden from 1990 to 2021. A: ASIR, B: ASPR, C: ASMR

From 1990 to 2021, the ASIR (AAPC = 0.83 [95% CI: 0.80 to 0.86]) and ASPR (0.68 [0.66 to 0.69]) of T1DM in AYAs globally showed an upward trend. Meanwhile, the ASMR (-0.35 [-0.56 to -0.14]) indicated a downward trend (Fig. 8). At the regional level, all regions had statistically significant (AAPC > 0 and *P* < 0.05) increases in ASIR and ASPR. The Western Pacific Region (1.71 [1.62 to 1.80]) experienced the largest increase in ASIR for T1DM in AYAs, while the European Region (1.55 [1.52 to 1.59]) had the largest increase in ASPR. The region with the largest increase in ASMR was the Region of the Americas (0.74 [0.51 to 0.97]), while the largest decrease occurred in the Western Pacific Region (-1.29 [-1.61 to -0.97]) (Table 2). On the national level, Cyprus (3.05 [2.93 to 3.16]) had the largest increase in ASIR for T1DM in AYAs, followed by Republic of Korea (2.88 [2.60 to 3.17]) and Argentina (2.88 [2.73 to 3.03]), while the largest decrease occurred in Maldives (-0.45 [-0.59 to -0.31]), followed by Saint Kitts and Nevis (-0.32 [-0.44 to -0.20]) and Thailand (-0.31 [-0.41 to -0.21]). Similarly, Cyprus (2.94 [2.89 to 2.98]) had the largest increase in ASPR, followed by the Ireland (2.85 [2.81 to 2.89]) and Spain (2.30 [AAPC = 2.07 to 2.52]), while the largest decreases were noticed in Cuba (-0.94 [-1.11 to -0.77]), Saint Kitts and Nevis (-0.85 [-0.95 to -0.76]), and Barbados (-0.76 [-0.82 to -0.70]). Regarding ASMR, Mexico (2.52 [2.09 to 2.96]) demonstrated the largest increase, followed by Zimbabwe (2.49 [2.09 to 2.89]) and Turkmenistan (2.42 [1.85 to 3.00]), while the largest decrease occurred in Singapore (-5.32 [-6.00 to -4.63]), Luxembourg (-4.54 [-5.22 to -3.86]), and Republic of Korea (-4.53 [-4.93 to -4.13]). Thirty-six countries had an increasing trend in ASMR(AAPC > 0 and *P* < 0.05). (Figs. 7.D, E, and F and Tables S7-9).

### Asthma

**Fig. 9:**
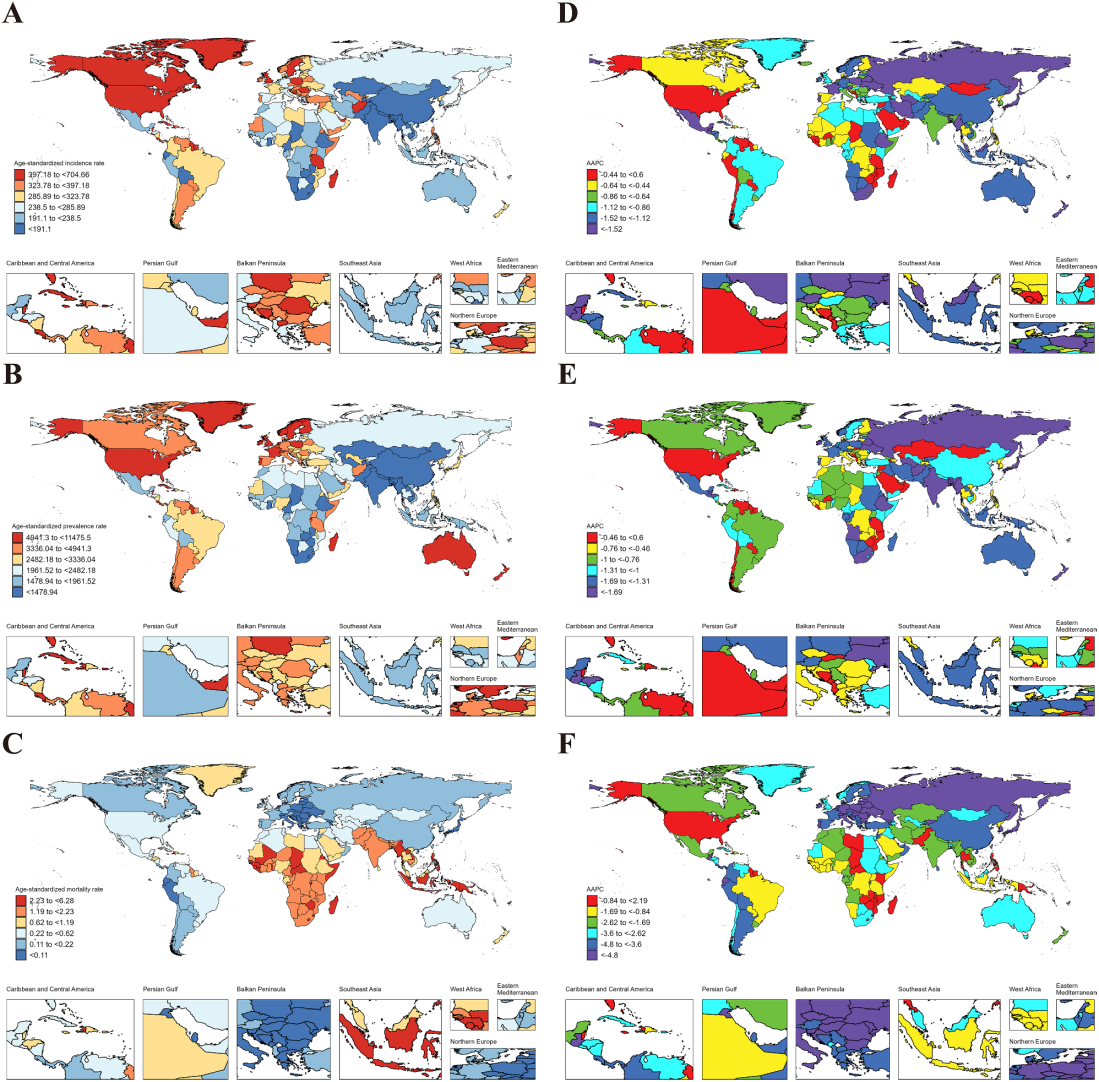
ASIR, ASPR and ASMR of Asthma in AYAs at the national level in 2021 (**A**, **B**, **C**), and their changing trends from 1990 to 2021 (**D**, **E**, **F**)

In 2021, the ASIR, ASPR, and ASMR of Asthma in global AYAs were 232.22 (95% UI: 132.11 to 361.24), 2245.51 (1671.05 to 2917.57), and 0.89 (0.77 to 1.08), respectively (Table 1). At the regional level, the Region of the Americas had the highest ASIR for Asthma in global AYAs (416.23 [222.15 to 674.32]), while the lowest ASIR (150.87 [88.73 to 232.94]) was observed in the South-East Asia Region. Similarly, Region of the Americas retained the top position for the highest ASPR (4340.40 [3177.27 to 5734.92]), and the South-East Asia Region (1233.42 [919.51 to 1598.10]) had the lowest ASPR. The region with the highest ASMR was the African Region (1.798 [1.404 to 2.429]), while the lowest ASMR was European Region (0.161 [0.150 to 0.176]) (Table 1). On the national level, the United States of America (704.66 [375.11 to 1138.21]) had the highest ASIR for Asthma in global AYAs, followed by United Arab Emirates (694.80 [389.51 to 1111.30]) and Haiti (641.69 [346.14 to 997.92]), while the lowest ASIR were in Nepal (87.46 [55.95 to 126.35]), Bhutan (95.59 [56.54 to 144.88]), and Bangladesh (103.46 [58.38 to 165.14]). Additionally, the United Kingdom had the highest ASPR for Asthma in AYAs (11475.50 [8546.43 to 14889.22]), followed by Portugal (9743.81 [6784.71 to 13047.20]) and Iceland (8095.78 [5667.93 to 10944.44]), while the lowest ASPR were in Nepal (643.77 [480.07 to 835.44]), Bhutan (789.94 [578.00 to 1050.99]), and Lesotho (832.21 [652.92 to 1043.44]) (Fig. 9. A, B, C & Table S4-6).

**Fig. 10:**
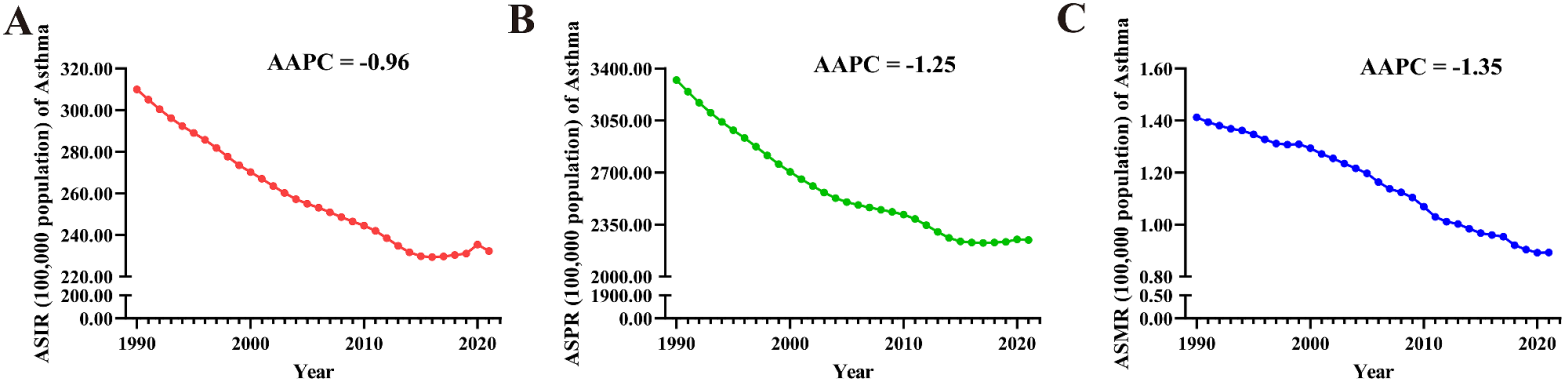
Trends in global Asthma burden from 1990 to 2021. A: ASIR, B: ASPR, C: ASMR

From 1990 to 2021, the ASIR (AAPC=-0.96 [95% CI: -1.03 to -0.88]), ASPR (AAPC=-1.25 [-1.31 to -1.19]), and ASMR (-1.35 [-1.44 to -1.26]) of Asthma in AYAs globally showed a significant decreasing trend (Fig. 10). At the regional level, the ASIR, ASPR, and ASMR of Asthma in AYAs demonstrated significant decreasing trends all regions (AAPC < 0 and *P* < 0.05), with the largest decreases observed in the European Region. At the national level, only the United States of America ‘s ASIR for Asthma in AYAs exhibits a statistically significant upward trend (AAPC = 0.79 [95% CI: 0.58 to 1.00]), while the largest decrease occurred in South Africa (-3.00 [-3.13 to -2.87]), followed by Singapore (-2.63 [-2.92 to -2.33]) and Russian Federation (-2.60 [-2.70 to -2.51]). The United States of America had the largest increase in ASPR for Asthma in AYAs (0.60 [0.43 to 0.78]), followed by Guyana (0.18 [0.14 to 0.23]), and Belize (0.04 [0.01 to 0.07]), while the largest decrease were noticed in New Zealand (-3.27 [-3.32 to -3.23]), Japan (-2.92 [-2.99 to -2.86]), and South Africa (-2.75 [-2.97 to -2.52]). The largest increase in ASMR was in Zimbabwe (2.19 [1.31 to 3.08]), followed by Lesotho (1.17 [0.89 to 1.45]), and Dominica (0.27 [0.06 to 0.47]), while the largest decrease occurred in Singapore (-9.50 [-11.01 to -7.97]), followed by Slovenia (-8.73 [-9.42 to -8.04]) and Japan (-8.56 [-9.42 to -7.70]). Only three countries (Zimbabwe, Lesotho, and Dominica) exhibited an upward trend in ASMR (AAPC > 0 and *P* < 0.05) (Fig. 9. D, E, F and Tables S7-9).

### Psoriasis

**Fig. 11:**
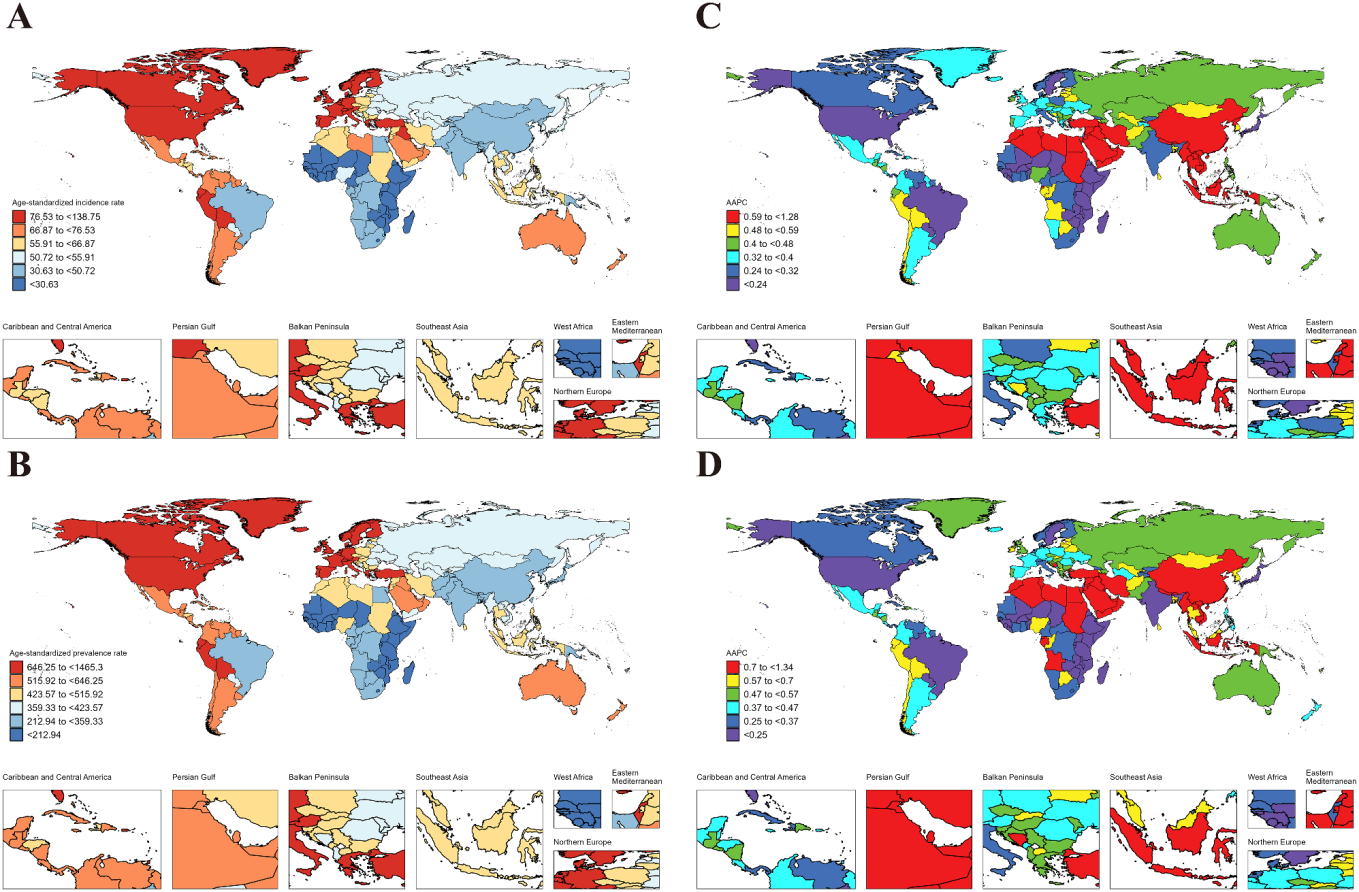
ASIR and ASPR of Psoriasis in AYAs at the national level in 2021 (**A**, **B**), and their changing trends from 1990 to 2021 (**C**, **D**)

In 2021, the ASIR and ASPR for Psoriasis in the global AYAs were 55.08 (95% UI: 48.53 to 61.93) and 426.16 (394.12 to 460.18), respectively (Table 1). At the regional level, the European Region exhibited the highest ASIR (82.19 [72.16 to 92.47]) and ASPR (725.76 [673.79 to 780.33]) for Psoriasis in global AYAs, while the lowest ASIR (33.44 [29.24 to 37.79]) and ASPR (260.36 [239.97 to 282.02]) were observed in the African Region (Table 1). On the national level, Germany (138.75 [120.13 to 159.85]) had the highest ASIR for Psoriasis in the global AYAs, followed by Switzerland (120.31 [104.55 to 137.03]) and Monaco (119.52 [104.15 to 135.55]), while the lowest ASIR were in Somalia (17.48 [15.37 to 19.71]), Rwanda (17.81 [15.33 to 20.54]), and Mozambique (19.64 [17.10 to 22.23]). Additionally, Germany (1465.30 [1356.40 to 1578.40]) had the highest ASPR for Psoriasis in the global AYAs, followed by Switzerland (1199.06 [1108.61 to 1293.25]) and Monaco (1190.68 [1102.97 to 1282.08]), while the lowest ASPR were Somalia (105.91 [96.69 to 115.62]), Rwanda (107.17 [96.16 to 118.81]), and Mozambique (121.95 [111.35 to 133.09]) (Fig. 11. A, B & Table S4-6).

**Fig. 12:**
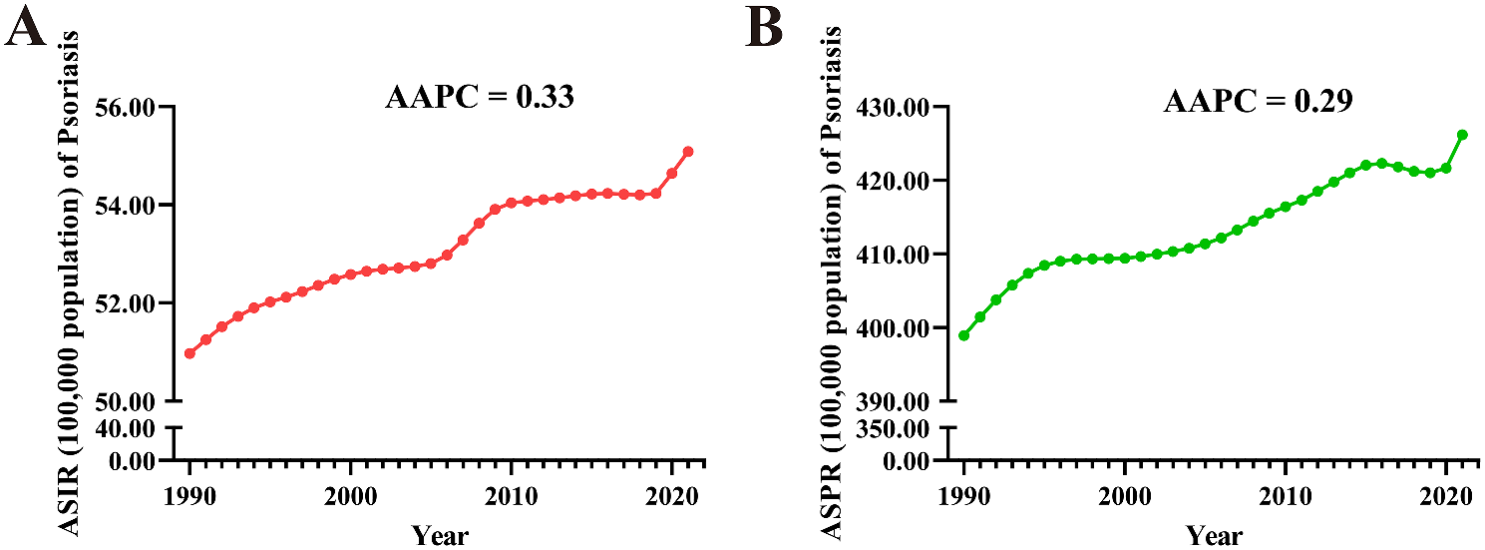
Trends in global Psoriasis burden from 1990 to 2021. A: ASIR, B: ASPR

From 1990 to 2021, the ASIR (AAPC = 0.33 [0.31 to 0.34]), ASPR (AAPC = 0.29 [0.27 to 0.32]) of Psoriasis in AYAs globally showed a significant increasing trend (Fig. 12). At the regional level, both ASIR and ASPR of Psoriasis in AYAs showed a significant increasing trend in all regions (AAPC > 0 and *P* < 0.05). The Western Pacific Region had the largest increase in ASIR (AAPC = 0.91 [0.86 to 0.97]) and ASPR (AAPC = 0.91 [0.89 to 0.94]). At the national level, the Maldives (1.28 [1.23 to 1.34]) had the largest increase in ASIR for Psoriasis in AYAs, followed by China (1.02 [0.96 to 1.07]) and Taiwan (Province of China) (1.00 [0.96 to 1.05]), while the largest decrease occurred in Somalia (-0.26 [-0.30 to -0.23]), followed by South Sudan (-0.14 [-0.16 to -0.11]) and Burundi (-0.10 [-0.13 to -0.08]). The Equatorial Guinea (1.34 [1.31 to 1.37]) had the largest increase in ASPR, followed by the Saudi Arabia (1.27 [1.26 to 1.28]) and Oman (1.22 [1.20 to 1.24]), while the largest decreases were noticed in Somalia (-0.33 [-0.36 to -0.29]), followed by South Sudan (-0.21 [-0.23 to -0.19]), and Burundi (-0.17 [-0.20 to -0.15]) (Fig. 11. C, D and Table S7-9).

### Variation of ASRs across regions and countries

#### Variation across regions

Based on our analysis, the ASIR, ASPR, and ASMR of major ADs in 2021 show variability across different regions. For ASIR, the greatest difference is observed in MS, with an EQ of 14.365 and a CV of 0.874, while the smallest difference is seen in RA, with an EQ of 2.847 and a CV of 0.358. For ASPR, the greatest difference is also in MS, with an EQ of 14.464 and a CV of 0.884, and the smallest difference in RA, with an EQ of 2.892 and a CV of 0.343. For ASMR, the greatest difference is in MS, with an EQ of 19.298 and a CV of 0.802, while the smallest difference is seen in T1DM, with an EQ of 2.939 and a CV of 0.324. (Supplementary Material-Table S10)

#### Variation across countries

In 2021, the comparison of major ADs across different countries reveals significant differences in ASIR, ASPR, and ASMR. For ASIR, the greatest difference is observed in IBD, with an EQ of 166.074 and a CV of 1.259, while the smallest difference is seen in asthma, with an EQ of 8.057 and a CV of 0.398. For ASPR, the greatest difference is also in IBD, with an EQ of 157.823 and a CV of 1.300, and the smallest difference in psoriasis, with an EQ of 13.835 and a CV of 0.572. For ASMR, the greatest difference is observed in RA, with an EQ of 179600.9 and a CV of 1.362, while the smallest difference is in T1DM, with an EQ of 55.1 and a CV of 0.678. (Supplementary Material-Table S11)

### Data supplementation

We provide “Burden of AD in adolescents (15–24 years, as defined by the World Health Organization [27, 28])” in the Supplementary Material (Table S12-16), which the reader is invited to review.

## Discussion

This study is the first to comprehensively analyze the burden of six major ADs—RA, IBD, MS, T1DM, Asthma, and Psoriasis—among AYAs in recent years. Utilizing data from the GBD 2021, we conducted a detailed examination of the ASIR, ASPR, and ASMR of these diseases globally and across various regions and countries. Additionally, we explored the trends in these rates from 1990 to 2021.

### Rheumatoid arthritis

RA shows an increasing trend in both ASIR and ASPR among AYAs, with a notable decline in ASMR. This indicates a growing chronic burden on this age group. Improved diagnostic capabilities and changes in environmental risk factors likely contribute to this trend. The impact of RA on quality of life and productivity due to chronic pain and joint deformity underscores the need for early diagnosis and long-term management strategies [29, 30]. This trend aligns with previous studies that have reported similar increases in RA incidence and prevalence among younger populations due to improved diagnostic capabilities and changing environmental risk factors [31, 32].

### Inflammatory bowel disease

IBD exhibits significant downward trends in both ASIR and ASPR globally, while the ASMR remains stable. The European Region has the highest rates, possibly due to genetic predispositions and environmental factors [33, 34]. In contrast, the lowest rates are observed in the African Region. The disruption caused by IBD in terms of appetite, weight loss, and fatigue highlights the necessity for early and effective interventions. This finding is consistent with studies that have shown higher IBD prevalence in Europe and North America compared to Africa and Asia [34].

### Multiple sclerosis

MS shows an upward trend in both ASIR and ASPR globally, with a decline in ASMR. The European Region has the highest incidence, prevalence, and mortality rates. MS diagnosed in AYAs often results in progressive neurological decline, significantly reducing life expectancy and quality of life [35, 36]. Continuous support and rehabilitation services are crucial to address these long-term health needs. According to Kingwell et al., MS is the most common cause of neurological disability in young adults worldwide and approximately half of those affected are in Europe [37]. Continuous support and rehabilitation services are essential to address the long-term health needs of AYAs with MS.

### Type 1 diabetes mellitus

T1DM presents the highest ASIR and ASPR among the ADs studied, with a significant upward trend globally. The European Region has the highest rates, while the Western Pacific Region has the lowest. T1DM poses lifelong challenges, including the risk of diabetic nephropathy and cardiovascular complications [38, 39]. Strengthening primary prevention and early intervention strategies is essential to manage this rising incidence and its long-term impacts. These results are in line with studies that highlight the high incidence of T1DM in Nordic countries and other parts of Europe [40], attributed to genetic factors and possibly environmental triggers [41].

### Asthma

Asthma demonstrates significant decreases in ASIR, ASPR, and ASMR globally, indicating effective public health interventions and management strategies [16]. The Region of the Americas shows the highest incidence and prevalence, while the African Region has the highest mortality. Continuous vigilance is necessary to sustain these gains and address emerging challenges. This trend of decreasing asthma incidence and prevalence has been documented in other studies, suggesting improvements in asthma management and public health policies globally [42].

### Psoriasis

Psoriasis shows consistent increases in both ASIR and ASPR globally, with significant regional differences. The highest rates are observed in Europe, indicating a higher burden of this disease. The chronic nature of psoriasis and its impact on physical and psychological health necessitate comprehensive care approaches to improve the quality of life for affected individuals [43, 44]. Previous literature corroborates these findings, showing that psoriasis prevalence is highest in Europe and North America, likely due to genetic factors and possibly lifestyle influences [42]. Addressing the psychosocial aspects and providing holistic management strategies are essential for improving the quality of life for affected individuals.

### Regional and national variations

This study highlights significant regional and national variations in the burden of ADs among AYAs. High-income regions, such as the European Region and the Region of the Americas, generally exhibited higher ASIR and ASPR. These higher rates can be attributed to better diagnostic capabilities and more advanced healthcare infrastructure [45]. However, these regions also showed lower ASMR, reflecting effective disease management and treatment protocols. In contrast, low- and middle-income regions, such as the African Region and the South-East Asia Region, often demonstrated lower ASIR and ASPR but higher ASMR, indicating potential gaps in healthcare access and quality. These disparities emphasize the need for tailored public health strategies and international collaboration to address the unique healthcare challenges and improve outcomes for AYAs in different regions.

### Variation of ASRs across regions and countries

Regionally, MS demonstrated the greatest difference in ASIR. This significant variability can be influenced by genetic predispositions and environmental factors such as latitude, particularly in Europe and North America, where healthcare systems are more advanced [46, 47]. On the other hand, RA exhibited the smallest difference in ASIR, indicating a more uniform global distribution of risk factors but still higher rates in high-income regions due to superior healthcare infrastructure and diagnostic capabilities [48].

Nationally, countries with well-developed healthcare systems, especially in Europe and North America, report higher ASIR and ASPR, reflecting better diagnostic and treatment facilities [49, 50]. Conversely, lower-income countries often show lower ASIR and ASPR but higher ASMR, highlighting disparities in healthcare access and quality [51]. These disparities underscore the need for tailored public health strategies and international collaboration to address these differences and improve outcomes for individuals with autoimmune diseases in different regions and countries.

### Limitations

Several limitations of this study should be noted. First, the GBD data relies heavily on modeling, which may introduce biases due to the variable quality of primary data from different countries. These biases can lead to inaccuracies in estimating the true burden of ADs. For instance, underreporting in low-income regions or overestimation in areas with better diagnostic facilities could skew the results. Second, the analysis did not include subgroup analyses, which could provide more detailed insights into the burden of ADs in specific populations. Subgroup analyses by gender, socioeconomic status, or specific age brackets within the AYA group could reveal more nuanced patterns and risk factors, aiding in the development of targeted interventions. Third, the variability in healthcare infrastructure and data collection methods across different regions and countries may affect the comparability of the results. Disparities in health system performance, disease surveillance, and reporting standards can introduce inconsistencies in the data, potentially leading to misinterpretations.

## Conclusion

ADs in AYAs present a significant public health challenge, with considerable variability in incidence, prevalence, and mortality rates across regions and countries. Understanding these patterns is crucial for developing targeted public health interventions and policies. This study provides valuable insights into the epidemiological landscape of ADs in AYAs, laying the groundwork for future research and improved healthcare strategies. Effective management of ADs in AYAs requires a comprehensive approach that includes early diagnosis, effective treatment, and ongoing support to improve long-term health outcomes.

### Electronic supplementary material

Below is the link to the electronic supplementary material.

Supplementary Material 1

## Data Availability

All data were obtained from the public open database: Global Health Data Exchange (GHDx) query tool (http://ghdx.healthdata.org/gbd-results-tool).

## Declarations

**Ethics**.

This research does not require ethical approval.

## Abbreviations

ASIR: age-standardized incidence rate
ASPR: age-standardized prevalence rate
ASMR: age-standardized mortality rate
ADs: Autoimmune diseases
AYA: Adolescents and young adults
RA: Rheumatoid arthritis
IBD: Inflammatory bowel disease
T1DM: Type 1 diabetes mellitus
AAPC: Average annual percentage change
CI: Confidence interval
ICD: International classification of diseases
UIs: Uncertainty intervals
CIs: Confidence intervals
EQ: Extremal quotient
CV: Coefficient of Variation

## References

[1] Gutierrez-Arcelus M, Rich SS, Raychaudhuri S. Autoimmune diseases - connecting risk alleles with molecular traits of the immune system. Nat Rev Genet. 2016;17:160–74. 10.1038/nrg.2015.33. 10.1038/nrg.2015.33 PMC4896831 26907721

[2] The global burden of adolescent and young adult cancer. In 2019: a systematic analysis for the global burden of Disease Study 2019. Lancet Oncol. 2022;23:27–52. 10.1016/s1470-2045(21)00581-7. 10.1016/S1470-2045(21)00581-7 PMC8716339 34871551

[3] Xie J, et al. Global burden of type 2 diabetes in adolescents and young adults, 1990–2019: systematic analysis of the global burden of Disease Study 2019. BMJ. 2022;379:e072385. 10.1136/bmj-2022-072385. 10.1136/bmj-2022-072385 PMC9727920 36740855

[4] Guan S-Y, et al. Global burden and risk factors of musculoskeletal disorders among adolescents and young adults in 204 countries and territories, 1990–2019. Autoimmun rev. 2023;22:103361. 10.1016/j.autrev.2023.103361. 10.1016/j.autrev.2023.103361 37230312

[5] Buzzetti R, Zampetti S, Maddaloni E. Adult-onset autoimmune diabetes: current knowledge and implications for management. Nat Rev Endocrinol. 2017;13:674–86. 10.1038/nrendo.2017.99. 10.1038/nrendo.2017.99 28885622

[6] Hua Y, et al. Phenotypes and genotypes of Chinese adult patients with systemic autoinflammatory diseases. Semin Arthritis Rheum. 2019;49:446–52. 10.1016/j.semarthrit.2019.05.002. 10.1016/j.semarthrit.2019.05.002 31155445

[7] Mohammedsaeed WM, Alghamdi ZJ. Autoimmune diseases and their prevalence in Saudi Arabian patients with type 1 diabetes mellitus. Saudi Med J. 2023;44:751–60. 10.15537/smj.2023.44.8.20230240. 10.15537/smj.2023.44.8.20230240 PMC10425616 37582563

[8] Venetsanopoulou AI, Alamanos Y, Voulgari PV, Drosos AA. Epidemiology of rheumatoid arthritis: genetic and environmental influences. Expert Rev Clin Immunol. 2022;18:923–31. 10.1080/1744666x.2022.2106970. 10.1080/1744666X.2022.2106970 35904251

[9] Ananthakrishnan AN. Epidemiology and risk factors for IBD. Nat Rev Gastroenterol Hepatol. 2015;12:205–17. 10.1038/nrgastro.2015.34. 10.1038/nrgastro.2015.34 25732745

[10] Parisi R, et al. National, regional, and worldwide epidemiology of psoriasis: systematic analysis and modelling study. BMJ. 2020;369(m1590). 10.1136/bmj.m1590. 10.1136/bmj.m1590 PMC7254147 32467098

[11] Baucom KJW, Turner SL, Tracy EL, Berg CA, Wiebe DJ. Depressive symptoms and diabetes management from late adolescence to emerging adulthood. Health Psychol. 2018;37:716–24. 10.1037/hea0000645. 10.1037/hea0000645 PMC6080212 30024228

[12] Solomon DH, et al. Cardiovascular morbidity and mortality in women diagnosed with rheumatoid arthritis. Circulation.2003;107:1303–7. 10.1161/01.cir.0000054612.26458.b2. 10.1161/01.cir.0000054612.26458.b2 12628952

[13] Shah SC, Itzkowitz SH. Colorectal Cancer in inflammatory bowel disease: mechanisms and management. Gastroenterology. 2022;162:715–e730713. 10.1053/j.gastro.2021.10.035. 10.1053/j.gastro.2021.10.035 PMC9003896 34757143

[14] Hirst C, Swingler R, Compston DA, Ben-Shlomo Y, Robertson NP. Survival and cause of death in multiple sclerosis: a prospective population-based study. J Neurol Neurosurg Psychiatry. 2008;79:1016–21. 10.1136/jnnp.2007.127332. 10.1136/jnnp.2007.127332 18303108

[15] Narayan KM, Boyle JP, Thompson TJ, Sorensen SW, Williamson DF. Lifetime risk for diabetes mellitus in the United States. JAMA. 2003;290:1884–90. 10.1001/jama.290.14.1884. 10.1001/jama.290.14.1884 14532317

[16] Beigelman A, Bacharier LB. Early-life respiratory infections and asthma development: role in disease pathogenesis and potential targets for disease prevention. Curr Opin Allergy Clin Immunol. 2016;16:172–8. 10.1097/aci.0000000000000244. 10.1097/ACI.0000000000000244 PMC5089840 26854761

[17] Takeshita J, et al. Psoriasis and comorbid diseases: Epidemiology. J Am Acad Dermatol. 2017;76:377–90. 10.1016/j.jaad.2016.07.064. 10.1016/j.jaad.2016.07.064 PMC5731650 28212759

[18] Global age-sex. -specific fertility, mortality, healthy life expectancy (HALE), and population estimates in 204 countries and territories, 1950–2019: a comprehensive demographic analysis for the global burden of Disease Study 2019. Lancet. 2020;396:1160–203. 10.1016/s0140-6736(20)30977-6. 10.1016/S0140-6736(20)30977-6 PMC7566045 33069325

[19] Global burden of 369 diseases and injuries in 204 countries and territories, 1990–2019: a systematic analysis for the Global Burden of Disease Study 2019. Lancet 396, 1204–1222. (2020). 10.1016/s0140-6736(20)30925-9. 10.1016/S0140-6736(20)30925-9 PMC7567026 33069326

[20] Measuring universal health coverage based on an index of effective coverage of health services. In 204 countries and territories, 1990–2019: a systematic analysis for the global burden of Disease Study 2019. Lancet. 2020;396:1250–84. 10.1016/s0140-6736(20)30750-9. 10.1016/S0140-6736(20)30750-9 PMC7562819 32861314

[21] Global. burden of 87 risk factors in 204 countries and territories, 1990–2019: a systematic analysis for the Global Burden of Disease Study 2019. *Lancet* 396, 1223–1249 (2020). 10.1016/s0140-6736(20)30752-2. 10.1016/S0140-6736(20)30752-2 PMC7566194 33069327

[22] Global regional. National disability-adjusted life-years (DALYs) for 315 diseases and injuries and healthy life expectancy (HALE), 1990–2015: a systematic analysis for the global burden of Disease Study 2015. Lancet. 2016;388:1603–58. 10.1016/s0140-6736(16)31460-x. 10.1016/S0140-6736(16)31460-X PMC5388857 27733283

[23] Boyle P, Parkin DM. Cancer registration: principles and methods. Statistical methods for registries. IARC Sci Publ, 126–58 (1991). 1894318

[24] Kim HJ, Fay MP, Feuer EJ, Midthune DN. Permutation tests for joinpoint regression with applications to cancer rates. Stat Med. 2000;19:335–51. 10.1002/(sici)1097-0258(20000215)19:3%3C335::aid-sim336%3E3.0.co;2-z. 10.1002/(sici)1097-0258(20000215)19:3<335::aid-sim336>3.0.co;2-z 10649300

[25] Woodward M. *Epidemiology: Study Design and Data Analysis, Third Edition*CRC Press,. (2013).

[26] Juran JM, Godfrey AB. Juran’s Quality Handbook. (McGraw Hill; 1999.

[27] Young Adult Health and Well-Being. A position Statement of the Society for Adolescent Health and Medicine. J Adolesc Health. 2017;60:758–9. 10.1016/j.jadohealth.2017.03.021. 10.1016/j.jadohealth.2017.03.021 28532650

[28] Economic UND o., Affairs S. *World Youth Report, 2007: Young People’s Transition to Adulthood: Progress and Challenges*United Nations, Department of Economic and Social Affairs,. (2007).

[29] Köhler BM, Günther J, Kaudewitz D, Lorenz H-M. Current therapeutic options in the treatment of rheumatoid arthritis. J Clin Med. 2019;8:938. 10.3390/jcm8070938 PMC6678427 31261785

[30] Gravallese EM, Firestein GS. Rheumatoid arthritis—common origins, divergent mechanisms. N Engl J Med. 2023;388:529–42. 10.1056/NEJMra2103726 36780677

[31] Kwok TSH, et al. Prevalence and factors Associated with osteoporosis and bone Mineral Density Testing in Psoriatic Arthritis. Arthritis Care Res. 2022;74:1006–12. 10.1002/acr.24538. 10.1002/acr.24538 33326187

[32] Li R, Yuan X, Ou Y. Global burden of rheumatoid arthritis among adolescents and young adults aged 10–24 years: a trend analysis study from 1990 to 2019. PLoS ONE. 2024;19:e0302140. 10.1371/journal.pone.0302140. 10.1371/journal.pone.0302140 PMC11020938 38625989

[33] Burisch J, et al. Environmental factors in a population-based inception cohort of inflammatory bowel disease patients in Europe — an ECCO-EpiCom study☆. J Crohn’s Colitis. 2014;8:607–16. 10.1016/j.crohns.2013.11.021. 10.1016/j.crohns.2013.11.021 24315795

[34] Ng SC, et al. Geographical variability and environmental risk factors in inflammatory bowel disease. Gut. 2013;62:630–49. 10.1136/gutjnl-2012-303661. 10.1136/gutjnl-2012-303661 23335431

[35] Dyment DA, Ebers GC, Sadovnick AD. Genetics of multiple sclerosis. Lancet Neurol. 2004;3:104–10. 10.1016/s1474-4422(03)00663-x. 10.1016/s1474-4422(03)00663-x 14747002

[36] Mazumder R, Murchison C, Bourdette D, Cameron M. Falls in people with multiple sclerosis compared with falls in healthy controls. PLoS ONE. 2014;9:e107620. 10.1371/journal.pone.0107620. 10.1371/journal.pone.0107620 PMC4177842 25254633

[37] Kingwell E, et al. Incidence and prevalence of multiple sclerosis in Europe: a systematic review. BMC Neurol. 2013;13:128. 10.1186/1471-2377-13-128. 10.1186/1471-2377-13-128 PMC3856596 24070256

[38] Liu Z, Wang H, Yang Z, Lu Y, Zou C. Causal associations between type 1 diabetes mellitus and cardiovascular diseases: a mendelian randomization study. Cardiovasc Diabetol. 2023;22:236. 10.1186/s12933-023-01974-6. 10.1186/s12933-023-01974-6 PMC10475187 37659996

[39] Teoh IH, Elisaus P, Schofield JD. Cardiovascular Risk Management in Type 1 diabetes. Curr Diab Rep. 2021;21:29. 10.1007/s11892-021-01400-9. 10.1007/s11892-021-01400-9 34448027

[40] Diaz-Valencia PA, Bougnères P, Valleron A-J. Global epidemiology of type 1 diabetes in young adults and adults: a systematic review. BMC Public Health. 2015;15:255. 10.1186/s12889-015-1591-y. 10.1186/s12889-015-1591-y PMC4381393 25849566

[41] Knip M, et al. Environmental Triggers and determinants of type 1 diabetes. Diabetes. 2005;54:S125–36. 10.2337/diabetes.54.suppl_2.S125. 10.2337/diabetes.54.suppl_2.s125 16306330

[42] Dharmage SC, Perret JL, Custovic A. Epidemiology of asthma in children and adults. Front Pead. 2019;7. 10.3389/fped.2019.00246. 10.3389/fped.2019.00246 PMC6591438 31275909

[43] Hepat A, Chakole S, Rannaware A. Psychological Well-Being of Adult Psoriasis patients: a narrative review. Cureus. 2023;15:e37702. 10.7759/cureus.37702. 10.7759/cureus.37702 PMC10191242 37206484

[44] Snast I, et al. Psychological stress and psoriasis: a systematic review and meta-analysis. Br J Dermatol. 2018;178:1044–55. 10.1111/bjd.16116. 10.1111/bjd.16116 29124739

[45] Finckh A, et al. Global epidemiology of rheumatoid arthritis. Nat Rev Rheumatol. 2022;18:591–602. 10.1038/s41584-022-00827-y. 10.1038/s41584-022-00827-y 36068354

[46] Goodin DS. In: Goodin DS, editor. Handbook of clinical neurology. Volume 122. Elsevier; 2014. pp. 231–66. 10.1016/B978-0-444-52001-2.00010-8 24507521

[47] Koch-Henriksen N, Sørensen PS. The changing demographic pattern of multiple sclerosis epidemiology. Lancet Neurol. 2010;9:520–32. 10.1016/S1474-4422(10)70064-8 20398859

[48] Almutairi K, Nossent J, Preen D, Keen H, Inderjeeth C. The global prevalence of rheumatoid arthritis: a meta-analysis based on a systematic review. Rheumatol Int. 2021;41:863–77. 10.1007/s00296-020-04731-0. 10.1007/s00296-020-04731-0 33175207

[49] Ghiam M, Gudis DA. in *Healthcare Disparities in Otolaryngology* 187–204Elsevier, (2024).

[50] Hernández-Negrín H, Roque-Dapresa Y, Martínez-Morales O, Mederos-Portal A. Using multiple cause-of-death analysis to estimate systemic autoimmune disease mortality burden in low-and middle-income countries. MEDICC Rev. 2021;23:69–74. 10.37757/MR2021.V23.N2.12 33974604

[51] Al-Worafi YM. *Handbook of Medical and Health Sciences in developing countries: education, practice, and research* (ed Yaser Mohammed Al-Worafi) 1–25. Springer International Publishing; 2023.

